# Design, Synthesis and Cytotoxicity Evaluationof New 1,2-diaryl-4, 5, 6, 7-Tetrahydro-1H-benzo[*d*] Imidazolesas Tubulin Inhibitors 

**Published:** 2015

**Authors:** Marzieh Amirmostofian, Farzad Kobarfard, Hamed Reihanfard, Vida Mashayekhi, Afshin Zarghi

**Affiliations:** a **Department of Medicinal Chemistry, School of Pharmacy, Shahid Beheshti University of Medical Sciences, Tehran, Iran. **; b **Department of Pharmaceutical Biotechnology, School of Pharmacy, Zanjan University of Medical Sciences, Zanjan, Iran.**

**Keywords:** 4, 5, 6, 7-Tetrahydro-1H-benzo[d]imidazole, Antitubulin, Molecular modeling, Cytotoxicity

## Abstract

A new series of 1,2-diaryl-4,5,6,7-tetrahydro-1*H*-benzo[d]imidazoles, possessing trimethoxyphenyl pharmacophore, were synthesized to evaluate their biological activities as tubulin inhibitors. Cytotoxic activity of the synthesized compounds 7a-f was assessed against several human cancer cell lines, including MCF-7 (breast cancer cell), HEPG2 (liver hepatocellular cells), A549 (adenocarcinomic human alveolar basal epithelial cells), T47D (Human ductal breast epithelial tumor cell line) and fibroblast. According to our results, HEPG2 seems to be the most sensitive, while MCF7 was the most resistant cell line to the compounds. All the compounds expect 7b, possessed satisfactory activity against HEPG2 with mean IC_50_ values ranging from 15.60 to 43.81 µM.

## Introduction

Based on WHO reports, cancer will be the first cause of death in the entire world in future (1,2). There is an intense effort in cancer research to develop novel compounds that are capable to stop cancer cell’s growth. Tubulins are rather new targets for cancer therapy, because of their crucial role in cellular functions such as cell shape maintenance, body structure of cilia flagella and immature blood vessels, transport and location of organelles (such as mitochondria, vesicles, *etc*.) and segregation of chromosome during mitosis (3-7).

Two globular tubulin heterodimer subunits (*α*-and *ß*-tubulin) alternately arrange to each other in a head-to-tail fashion to form a string which is called protofilament. Twisting around a hypothetical axis, usually 13 protofilaments constitute a hallow microtubule cylinder (8-13), which as mentioned, has a key role in regulation process.

There are some antimitoic agents which act by targeting three sections of *ß*-tubulin. These sections, thus, was named based on the interaction of the different classes of antimitoic agents with their binding sites on tubulin. The binding regions discussed are vinca, taxol and colchicine sites (14-16).

**Figure 1:**
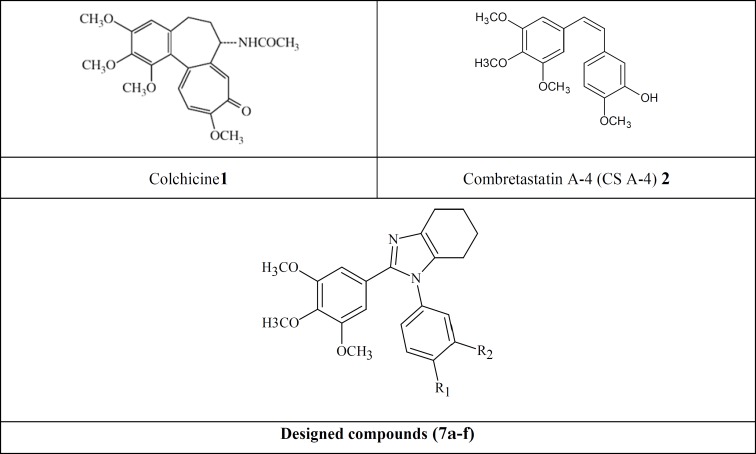
Tubulin inhibitors, lead compounds (CS A-4 and Colchicine), and our designed scaffold.

Colchicine (Figure 1, 1) is one of the earliest microtubule-targeting agents which were isolated from the meadow saffron *colchicium autumnale*. In fact tubulin was first purified based on its high affinity binding with colchicine and was named as a colchicine-binding proteins (17-21). Because of its high adverse effects, the clinical development of colchicine for cancer treatment has not been successful (22). The therapeutic value of colchicine is well established in the treatment of acute gouty arthritis, familial Mediterranean fever (FMF), Behcet’s disease and recurring pericarditis with effusion (21,23,24).

**Figure 2:**
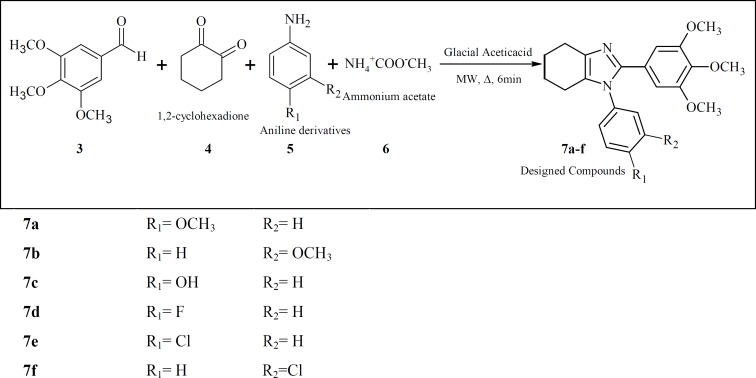
Synthesis of compounds 7a-h

In 1989, Pettit and his co-workers isolated combretastatin (CA-4) from the African willow tree, *combretumcaffrum *(25). CA-4 (Figure 1, 2) has a great antitumor effect by binding to colchicine binding site on ß-tubulin. It is capable of rapid and selective disruption of the abnormal tumor vasculature and also exhibits potent cytotoxicity against a board spectrum of cancer lines, including those that are multi-drug resistance (MDR) (26-28). Due to the poor aqueous solubility and bioavailability of CA-4(29),a wide range of structural analogs of CA-4 have been synthesized and structure activity relationship (SAR) has been determined for these derivatives. According to the existing SAR for CA-4, the 3,4,5-trimethoxyphenyl ring (A ring),*cis*-configuration of the two phenyl rings, two- atom distance between them and a phenyl ring (B ring)with H-donor groups on positions 3 or 4 are essential for optimum activity (30).

In our study, a series of new analogs are reported that in their structures ring A and B are connected to each other in *cis* geometry via a tetrahydrobenzimidazole ring (Figure 1, compounds 7a-f). Docking simulation was performed to position these compounds into colchicine binding site to determine the probable binding model, which suggested the possible inhibition mechanism. Cytotoxic activity of the synthesized compound was also studied against MCF-7, HEPG2, A549, T47D and normal fibroblast cells.

## Experimental

*Chemicals*

All chemicals, reagents and solvents used in this study were purchased from Merck AG and Aldrich Chemical Company and were used without further purifications. Melting points were determined with a Thomas–Hoover capillary apparatus. Infrared spectra were acquired using a Perkin Elmer Model 1420 spectrometer. A Brucker FT-500 MHz instrument (Brucker Biosciences, Germany) was used to acquire 1HNMR spectra with TMS as internal standard. Chloroform-D and DMSO-D_6_ were used as solvents. Coupling constants (*J*) values are calculated in hertz (Hz) and spin multiplicities are given as s (singlet), d (double), t (triplet) and m (multi). The mass spectral measurements were performed on a 6410Agilent triple quadrupole mass spectrometer (LCMS) with an electrospray ionization (ESI) interface.

*Chemistry*

*General procedure for the preparation of compounds 7a-f*

Amixture of 3,4,5-trimethoxyaldehyde (3, 2 mmol), 1,2-cyclohexadione (4, 2 mmol), an aniline derivatives (5, 2.4 mmol) and ammonium acetate(6,2 mmol) in15 mL glacial acetic acid was placed in a 20 mL microwave reactor and stirred under microwave irradiation (150 W) at approximately 120 oC for 6 minutes. The completion of the reaction was monitored by TLC. The reaction mixture was then concentrated under reduced pressure in a water bath. The dark sticky residue was subjected to a column chromatography using a 30 cm silica gel column as stationary phase and Ethyl actate/Hexan (5:1) as mobile phase to obtain the purified compounds 7a-f.

*1-(4-Methoxyphenyl)-2-(3,4,5-trimethoxyphenyl)-4,5,6,7-tetrahydro-1H-benzoimidazole (7a)*

Yield,14%;brown powder; mp 110-111 oC; IR (KBr disk): γ (cm-1) 1578 (C=N), 1600-1400 (aromatic), 1130 (C-N);LC-MS (ESI) *m/z*: 395(M+1, 100).1HNMR (CDCl_3_, 500 MHz): δ 1.85-1.91 (m, 4H,H_5,6 _of cyclohexyl ring), 2.38-2.40(t, 2H, H_4_of cyclohexyl ring,* J*=5.74, 6.09 Hz), 2.75-2.78 (t, 2H, H_7_ of cyclohexyl ring,* J*=5.7, 6.1 Hz),3.66 (s, 6H, O-CH_3 _of ring A), 3.84 (s, 3H, O-CH_3_ of ring A), 3.86 (s, 3H, O-CH_3_ of ring B), 6.65 (s, 2H, H_2,6_ of ring A), 6.97-6.98 (d, 2H, H_3,5_ of ring B,* J*=5 Hz), 7.16-7.17 (d, 2H, H_2,6_ of ring B,*J*=5 Hz).

*1-(3-Methoxyphenyl)-2-(3,4,5-trimethoxyphenyl)-4,5,6,7-tetrahydro-1H-benzoimidazole (7b)*

Yield,10%;red liquid; IR (KBr disk): γ (cm-1) 1596 (C=N), 1600-1400 (aromatic), 1141 (C-N); LC-MS (ESI) *m/z*: 395(M+1, 100).1HNMR (CDCl_3_, 500 MHz): δ1.82-1.87 (m, 4H, H_5,6_of cyclohexyl ring), 2.39 and 2.72 (two broad singlet, 4H, H_4,7_ of cyclohexyl ring),3.61 (s, 6H, O-CH_3 _of ring A), 3.75 (s, 3H, O-CH_3_ of ring A), 3.80 (s, 3H, O-CH_3_ of ring B), 6.62 (s, 2H, H_2,6_ of ring A), 6.73 (s,1H, H_2_ of ring B), 6.80-6.82 (d,1H, H_4_ of ring B, *J*=7.6 Hz), 6.92-6.94 (d,1H, H_6_ of ring B, *J*=8.4 Hz), 7.32-7.35 (t,1H, H_5_ of ring B, *J*=7.96, 8.04 Hz).

*4-[2-(3,4,5-Trimethoxyphenyl)-4,5,6,7-tetrahydro-1H-benzoimidazole-1-yl]-phenol (7c)*

Yield: 15.3%, off-white powder; mp: 259-260 oC; IR (KBr disk): γ (cm-1) 3383 (OH), 1600-1400 (aromatic), 1223 (C-N); LC-MS (ESI)*m/z*: 381(M+1, 100).1HNMR (CDCl_3_, 500 MHz): δ 1.75 (m, 4H, H_5,6_of cyclohexyl ring), 2.28 and 2.55 (two broad singlet, 4H, H_4,7_ of cyclohexyl ring), 3.53 (s, 6H, O-CH_3 _of ring A), 3.61 (s, 3H, O-CH_3 _of ring A), 6.58 (s, 2H, H_2,6_ of ring A), 6.85-6.87 (d, 2H,H_3,5_ of ring B, *J*=8.68 Hz), 7.08-7.09 (d, 2H,H_2,6_ of ring B, *J*=8.7 Hz),8.3 (s, 1H, OH).

*1-(4-Fluorophenyl)-2-(3,4,5-trimethoxyphenyl)-4,5,6,7-tetrahydro-1H-benzoimidazole (7d)*

Yield,23.3%;pink powder; mp 152-154 oC; IR (KBr disk): γ (cm-1) 1685 (C=N), 1600-1400 (aromatic); LC-MS (ESI) *m/z*: 383(M+1, 100).1H NMR (CDCl_3_, 500 MHz): δ 1.81-1.83 (m,4H, H_5,6 _of cyclohexyl ring), 2.33 and 2.69 (two broad singlet, 4H, H_4,7_of cyclohexyl ring),3.60 (s, 6H, O-CH_3 _of ring A), 3.78 (s, 3H, O-CH_3_ of ring A), 6.54 (s, 2H, H_2,6_ of ring A), 7.15-7.16 (d, 2H, H_2,6_ of ring B, *J*=4.67 Hz), 7.09-7.13 (t, 2H,H_3,5_ of ring B, *J*=8.2, 8.5 Hz). 

*1-(4-Chlorophenyl)-2-(3,4,5-trimethoxyphenyl)-4,5,6,7-tetrahydro-1H-benzoimidazole (7e)*

Yield, 24%;brown powder; mp 157-158 oC; IR (KBr disk): γ (cm-1) 1584 (C=N), 1600-1400 (aromatic), 1135 (C-N); LC-MS (ESI) *m/z*: 399,401 (M+1, 100). 1HNMR (CDCl_3_, 500 MHz): δ 1.86-1.90 (m, 4H, H_5,6_of cyclohexyl ring), 2.41 and 2.76 (two broad singlet, 4H, H_4,7_of cyclohexyl ring),3.66 (s, 6H, O-CH_3 _of ring A), 3.85 (s, 3H, O-CH_3_ of ring A), 6.59 (s, 2H, H_2,6_ of ring A), 7.17-7.19 (d, 2H, H_2,6_ of ring B, *J*=8.6 Hz), 7.44-7.46 (d, 2H, H_3,5_ of ring B,*J*=8.6 Hz).

*1-(3-Chlorophenyl)-2-(3,4,5-trimethoxyphenyl)-4,5,6,7-tetrahydro-1H-benzoimidazole (7f)*

Yield, 16%; yellow powder; mp289-290 oC; IR (KBr disk): γ (cm-1) 1586 (C=N), 1600-1400 (aromatic), 1130 (C-N); LC-MS (ESI) *m/z*: 399,401 (M+1, 100). 1H NMR (CDCl_3_, 500 MHz): δ 1.87-1.92 (m, 4H, H_5,6_of cyclohexyl ring), 2.43 and 2.76 (two broad singlet, 4H, H_4,7_of cyclohexyl ring),3.67 (s, 6H, O-CH_3 _of ring A), 3.87(s, 3H, O-CH_3 _of ring A), 6.61 (s, 2H, H_2,6_ of ring A), 7.10-7.14 (m, 2H, two aromatic hydrogens of ring B), 7.41 -7.44 (m, 2H, two aromatic hydrogens of ring B).

*Anticancer activity*

Fibroblast, MCF7 (human breast cancer), HEPG2 (human liver carcinoma), A549 (human lung cancer) and T47D (human breast cancer) cell lines were purchased from Pasteur Institute, Tehran, Iran. The cells were grown in RPMI1640 medium at 37 °C under 5% CO_2_ supplemented with 10% heat inactivated fetal bovine serum (FBS), 100 U/mL penicillin and 100 µg/mL streptomycin. Cell viability was evaluated using MTT method which is based on the reduction of 3-(4,5-dimethylthiazol-2-yl)-2,5-diphenyltetrazolium bromide (MTT) dye to purple formazan crystals by mitochondrial succinate dehydrogenase enzyme in living cells. The cells were seeded into 96-well plates at a concentration of 6000 cells/well and allowed to incubate for 24 h. The medium was then discarded and different concentrations of the test compounds dissolved in complete medium, were added to each well. After further incubation for 24 h at 37 °C, the medium was aspirated and 100 µL MTT (2 mg/mL) was added to the wells and incubated for 3 h at 37 °C. The produced formazan crystals were dissolved in 100 µL of DMSO. Plates were incubated for 20 min at 37 °C and the optical densities were read at 570 nm with a reference wavelength of 630 nm as background using a spectrophotometer plate reader (Infinite® M200, TECAN). Colchicine was used as positive control and DMSO as the solvent of the test compounds and its final concentration was less than 0.2%. IC_50_ was calculated by calibration curve using Prism software. All the tests were performed in triplicates.

*Molecular modeling (docking) studies*

**Figure3:**
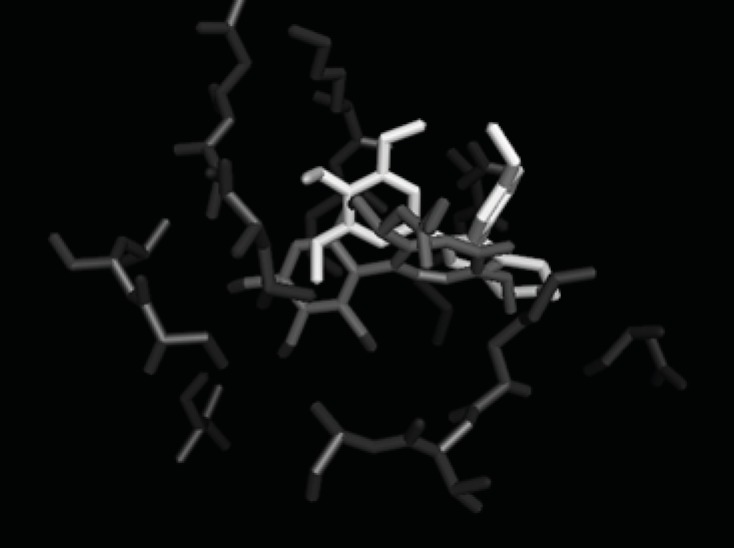
Molecular Modeling: good superimposition of the trimethoxyphenyl moiety of the synthesized compound 7a with the corresponding ring of colchicine.

All the synthesized compounds were subjected to molecular modeling study and their interactions with the colchicine binding site of β-tubulin were simulated. The tubulin structure was downloaded from the PDB data bank (http://www.rcsb.org/ - PDB code: 1SA0) (31, 32). All the compounds were built using Chem Draw software and minimized subsequently. The protein structure was prepared for docking using AUTODOCK Tool. Docking was performed by AutoDock 4.2 program using the implemented empirical free energy function and the Lamarckian genetic algorithm (LGA) (32). Crystallized ligand was removed from crystal protein (1SA0). Polar hydrogens were added and non-polar hydrogens were merged and finally Kollman united atom charge and atom type parameters were added to 1SA0. Grid map dimensions (126×126×126) were set surrounding active site. Lamarckian genetic search algorithm was employed and docking run was set to 50. The results showed good superimposition of the trimethoxyphenyl moiety of the synthesized compound 7a with the corresponding group of colchicine (Figure 3).

## Results and Discussion

The target 4,5,6,7-tetrahydro-1*H*-benzo[d]imidazole derivatives were synthesized based on the synthetic procedure described previously by Zarghi *et al. *(33). Microwave accelerates the reaction of 3,4,5-trimethoxybenzaldehyde (1) with 1,2-cyclohexadione (2) and excess of different substituted aniline (3) in the presence of glacial acetic acid and ammonium acetate to form compounds 7a-f. All the compounds were confirmed using IR, ESI–MS and 1HNMR spectroscopies. Yields of the resulting compounds 7a-f ranged from 10% to 24%. 

Cytotoxic activity of the synthesized compounds 7a-f was assessed against several human cancer cell lines, including MCF-7 (breast cancer cell), HEPG2 (liver hepatocellular cells), A549 (adenocarcinomic human alveolar basal epithelial cells), T47D (Human ductal breast epithelial tumor cell line) and fibroblast following a previously described procedure (34). The results are shown in Table 1. The comparative cytotoxic activities of the synthesized compounds against Fibroblast, MCF7, HEPG2, A549 and T47D indicate difference in responsiveness/sensitivity of different cancerous cells. In general HEPG2 seems to be the most sensitive, while MCF7 was the most resistant cell line to the compounds. All the compounds expect 7b, possessed satisfactory activity against HEPG2 with mean IC_50_ values ranging from 15.60 to 43.81 µM. 

Compound 7e exhibited the most potent activities against HEPG2. Selectivity of the cytotoxic activity of the six synthesized compounds was assessed by comparing the cytotoxic activity (IC_50_) of each compound against each cancerous cell with that of the normal human Fibroblast cells (Table 1). Results were expressed as selectivity index (SI). Compounds 7c, 7d and 7e appear to be the most selective against HEPG2. The highest selectivity was, however, observed for compound 7c against HEPG2 cell line (SI~ 4.1).

Molecular modeling studies comparing colchicine and the synthesized compound 7a were carried out. The structures were superimposed by a least square fit of the 3,4,5-trimethoxyphenyl moieties. The results are shown in Figure 3. As anticipated, the crucial orientation and spacing of the two aromatic rings were essentially retained in the new compounds. The bridge moieties cause the A ring and B ring of these compounds to lie in a similar positions to that of the colchicine site. 

**Table 1:** *In* *-* *vitro* antiproliferative activity of compounds 7a-f based on MTT assay and their selectivity index (SI).

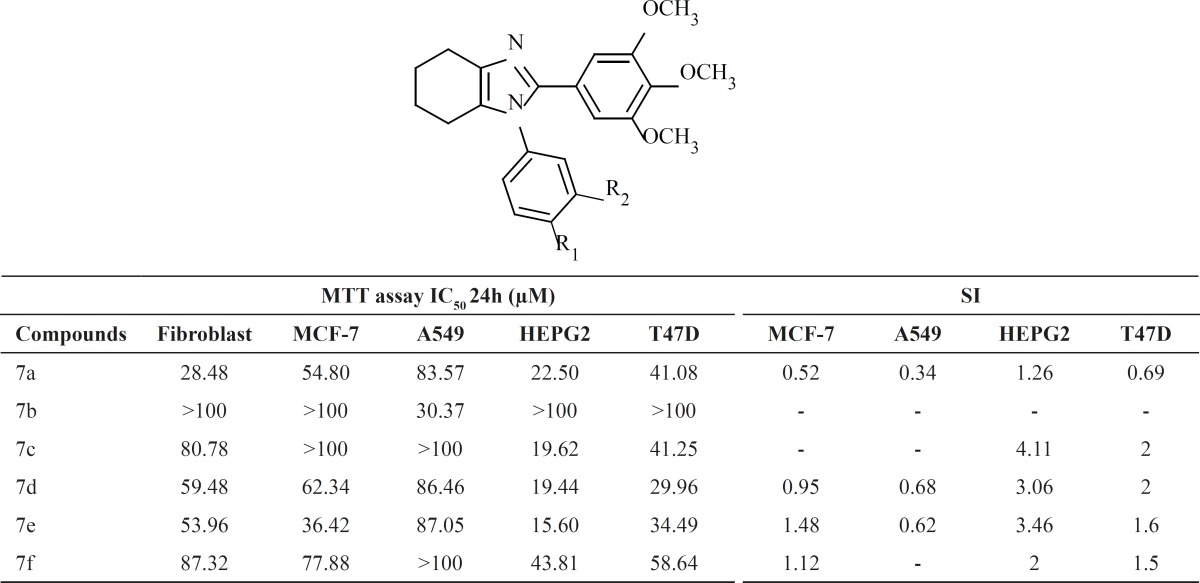

IC_50_: drug concentration that inhibits cell growth by 50%.

SI was calculated by dividingIC_50_ value against Fibroblast for each compound by the IC_50_value of that compound against the cancer cell line.
